# Program evaluation of the Janani Suraksha Yojna

**DOI:** 10.1186/1753-6561-6-S5-O15

**Published:** 2012-09-28

**Authors:** Rajani Ved, Thiagarajan Sundararaman, Garima Gupta, Geetha Rana

**Affiliations:** 1 National Health System Resource Centre, Delhi, India; 2 Department for International Development, Patna, India

## Introduction

The Janani Suraksha Yojana (JSY) was launched in 2005 as a response to high maternal mortality in India. The scheme was driven by global evidence that conditional cash transfers enable behaviour change, in this case, rise in institutional delivery. The scheme had a differential incentive entitlement based on urban or rural areas and high or low performing states. The incentives for women in rural areas of low performing states are: INR 1400 for institutional delivery and INR 500 for home deliveries. However in case of home deliveries many exclusionary practices denied benefits of the scheme to women below 19 years of age, higher parity and those entitled but without holding the Below Poverty Line (BPL) card.

The paper draws on a study evaluating the effectiveness of the scheme. The objectives of the evaluation were: to assess trends in institutional delivery, the availability and quality of care at delivery and in post natal period; the capability of health institutions and the role of village level health workers called the Accredited Social Health Activists (ASHA). Impact evaluation on maternal mortality was not attempted.

## Methods

Three districts in each of eight EAG (Empowered Action Group) states were selected as high performing, poor performing and tribal districts. This categorisation was based on number of institutional deliveries during 2008-09 and proportion of scheduled caste/ tribe population. Quantitative and qualitative methods were used to map the contexts, mechanisms and outcomes as evidenced from primary and secondary data. The quantitative survey conducted in twelve districts included 2759 institutional deliveries and 710 home deliveries. In addition to assessing access and quality of services, the evaluation aimed to capture what actually happened and why.

## Results and discussion

The study shows that over 50% of women who had their previous delivery at home had opted for institutional delivery. However despite this increase, there were about 40% home deliveries, with a range from 7.7% to 63%. Reasons reported for home delivery included limited access to transport, poor service quality, high costs in institutions and cultural preferences.

Home deliveries were primarily occurring among women who were younger than 19 years, had higher parity, belonged to marginalized groups and without the entitlement card that would provide preferential access. The study also demonstrated significant out of pocket expenses. Such expenses amounted to INR 1400 to INR 1600 on an average, primarily on account of payment for transport and drugs. The steep increase in institutional delivery, despite the fact that out of pocket expenditure exceeded the cash transfer, signified that women preferred institutional delivery for health and safety reasons. The increased availability of services was also a major contributor to the change. Private sector accounted for only about 12.5% of all deliveries. However, for complications, private sector provided 60% of care though for complications that required hospitalization, the distribution between public and private sector was almost equal.

The study supports the contention that JSY has resulted in an increase in institutional deliveries, and that it has enabled and empowered poor women to access public health facilities. However issues like the exclusionary criteria for both home deliveries limit access of the most vulnerable category. Persistent high out of pocket payments also need to be addressed urgently. Increases in human resources and infrastructure had not been sufficient to provide quality services. Major recommendations emphasized, removal of all exclusionary criteria in case of home and institutional deliveries, provision of free pregnancy and newborn care and increasing infrastructure and human resource providing emergency obstetrics and neonatal care services.

The interpretation of findings and recommendations in the emerging context of universal health care, resulted in converting a scheme based on the logic of conditional cash transfer into an “enabling entitlement” approach. Emphasis shifted to assured free drugs and transport to pregnant, post partum women and newborns and the pressure builds up to remove all conditionalities.

## Competing interests

Authors declare that they have no conflict of interest.

## Funding statement

This study was funded by the Ministry of Health and Family Welfare, Government of India.

